# Development of indicators for monitoring Community-Based Rehabilitation

**DOI:** 10.1371/journal.pone.0178418

**Published:** 2017-06-02

**Authors:** Catherine Mason, Joerg Weber, Seryan Atasoy, Carla Sabariego, Alarcos Cieza

**Affiliations:** 1 Department of Medical Informatics, Biometry and Epidemiology – IBE, Public Health and Health Services Research, Ludwig-Maximilians-University (LMU), Munich, Germany; 2 International Centre for Evidence in Disability, London School of Hygiene and Tropical Medicine, London, United Kingdom; 3 Disability and Rehabilitation Unit, World Health Organization, Geneva, Switzerland; IRCCS E. Medea, ITALY

## Abstract

**Background:**

Community-Based Rehabilitation (CBR) is a multi-sectoral approach working to equalize opportunities and include people with disability in all aspects of community life. Reliable and internationally comparable data needed to monitor and evaluate CBR are scarce, partially due to the absence of standardized indicators. The objective of this manuscript is to describe the collaborative development process which led to the World Health Organization's (WHO) recently launched set of standardized CBR outcome indicators.

**Methods:**

The WHO's CBR Guidelines recognize CBR as a comprehensive and multi-sectoral strategy, and were therefore used as the starting point for the development of the indicators, in a consensus process involving WHO and International Disability and Development Consortium. Pilot implementations in Guatemala, Egypt and China using a specifically developed mobile phone application to collect data, and an online expert survey were completed to assess validity and feasibility of the indicators and their corresponding questions.

**Results:**

The indicator set includes 13 Base Indicators which are broad enough to capture the situation of people with disability in settings where CBR is carried out, independently of the specific CBR activities carried out in a community; and 27 Supplementary Indicators that provide more specific coverage and can be selected based on the specific goals of a CBR program.

**Conclusion:**

The indicators were suitable to assess differences in health, education, social life, livelihood and empowerment between people with disability and other community members. This comparability provides valuable information to CBR managers, donors and government agencies, to guide decision making, support advocacy and improve accountability. The CBR indicators will support WHO and its member states in their efforts towards strengthening CBR, by generating evidence on its effectiveness.

## Introduction

**Community-based Rehabilitation (CBR) is an umbrella-term for strategies “within general community development for rehabilitation, equalization of opportunities, and social inclusion of all people with disabilities” that aim to address their wider needs in their communities.** CBR is implemented through the combined efforts of people with disability themselves, their families and communities, and the relevant service sectors[1]. CBR is implemented in over 100 countries, evolving from its initial focus on limitations and barriers experienced in low-and middle-income countries to also be relevant for higher-income countries[2,3]. However, CBR coverage is usually very low regarding the proportion of people with disability receiving support, CBR is seldom integrated into health or social security systems, and is instead usually financed and provided by non-governmental organizations (NGOs)[4]. Acknowledging the importance of CBR in tackling stigma, discrimination, barriers to equal participation, and lack of appropriate services faced by people with disability[5], the World Health Organization (WHO) set strengthening CBR, particularly through fostering the improvement of CBR monitoring and evaluation, as one objective of the recently endorsed *Global Disability Action Plan*[6].

**Sound and systematic CBR monitoring and evaluation is a significant challenge faced by the CBR sector in promoting and advocating for its broader implementation.** While anecdotal evidence exists on the success of CBR, internationally comparable results are still rare, and reliable and comparable data needed to monitor and evaluate CBR scarce[4,7,8]. Although the existing qualitative work delivers essential in-depth understanding of the changes CBR initiates[9], the lack of standardized measures limits the generated evidence and the comparability across settings[7,10]. A recently published systematic review reporting evidence on the effectiveness of CBR in low- and middle-income countries pointed out promising results in terms of clinical outcomes, functioning and quality of life, but could not deliver clear evidence due to the heterogeneity of interventions and quality of included studies[4]. A second review examining the methods used to collect data on CBR programs corroborates the lack of standardisation. This particular review calls for the development of a data collection method which takes the complexity and heterogeneity of CBR into consideration while keeping a high level of standardisation[7].

**Fig 1:**
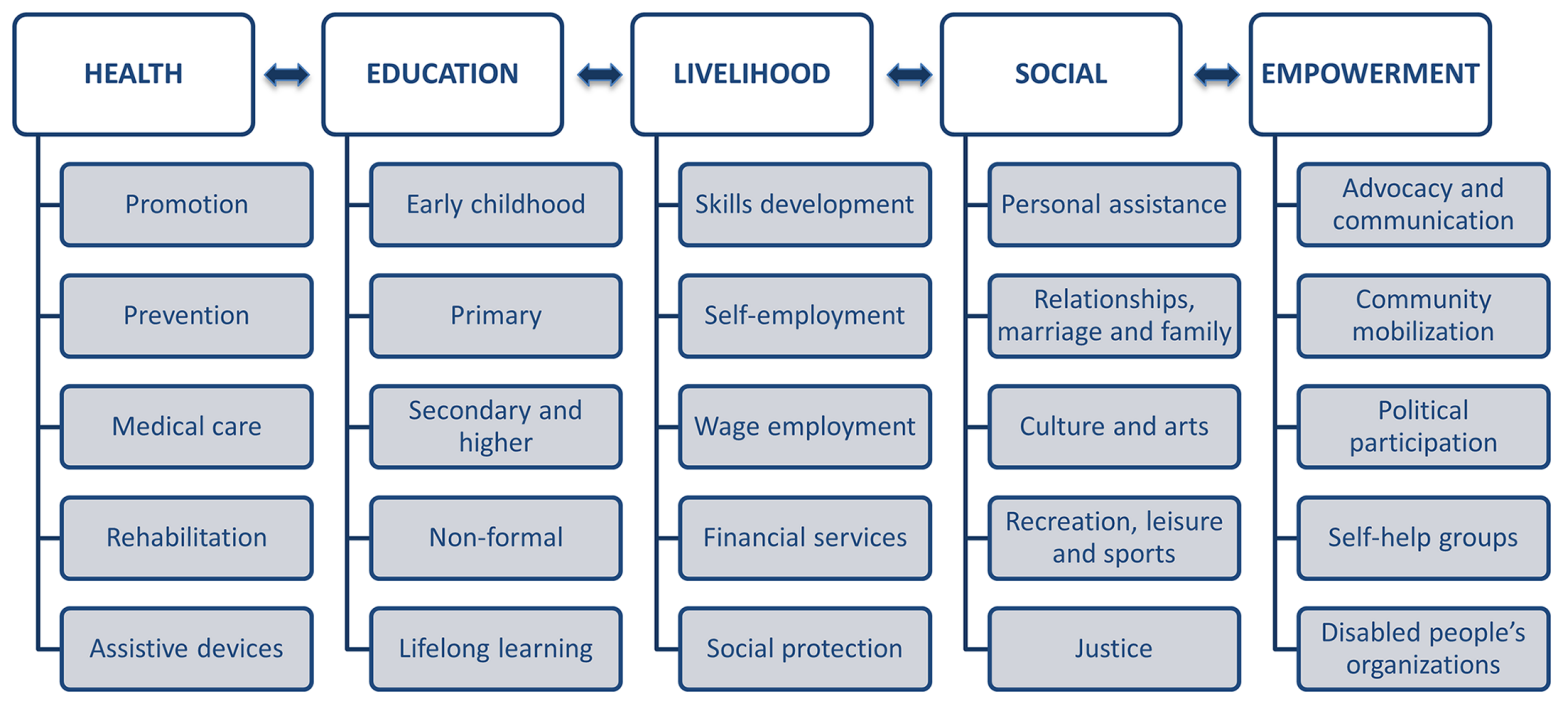
The CBR Matrix.

**Indeed, several attempts have been taken towards developing standardized data collection methods for CBR, by attempting to identify reoccurring CBR domains, to suggest evaluation frameworks, and to develop specific indicators**. In 1995, a joint WHO workshop looked to develop outcome indicators with the goal of providing qualitative information about the effectiveness of CBR activities, with a special effort to create indicators beyond the health component of CBR[11]. One of the first attempts to introduce the use of classification models to evaluate CBR was suggested in 2000, which used four dimensions with a defined scoring system to categorize programs[12]. A few years later, Wirz and Thomas noted that many studies have attempted to compile sets of indicators to judge the effectiveness of CBR. Based on ten included studies, they identified six activity domains and derived indicators in line with these activities[13]. One year later, a workshop developed a template that comprised of a number of guiding questions within three domains, which were then later developed into a set of evaluation indicators[14]. In 2010, the release of the WHO’s Community-Based Rehabilitation Guidelines (hereafter CBR Guidelines) served to synthesize global perspectives on CBR, and have since become accepted internationally as a conceptual framework for CBR[15].With these guidelines, the WHO recognized that no single model of CBR is appropriate for the whole world and suggested the pre-existing CBR Matrix (Fig 1) as a common framework to reflect the comprehensive multi-sectoral strategy that is CBR. In 2012 the CBR Guidelines were used during a WHO technical meeting[16] as a guide to develop a set of indicators, focusing mainly on access to CBR services, and being in this sense restricted to a single perspective. Furthermore, consensus was not reached regarding these indicators, and they were therefore not promoted for use. A CBR Monitoring Manual and Menu, published in 2015, outlines possible methods, encourages the setup of easy and routine monitoring and provides information that can be used as building blocks for indicators. However, standardized indicators are not presented[17].

**Despite these efforts towards standardization for monitoring CBR, a standardized multi-sectoral and internationally comparable set of CBR indicators suitable to monitor the changes that different CBR strategies initiate in the lives of people with disability, is lacking**[4]. Previous CBR indicators, however, tend to describe practices and stakeholder perceptions, rather than asses the changes brought by these practices[13]. Furthermore, they tend to target specific CBR activities or are tailored for a specific region[14,18,19]. Indicators suitable to monitor CBR across communities and countries need to be generic in that they can be used to monitor CBR independent of the specific objectives and activities of individual CBR programs. Also absent from previous CBR indicator initiatives is the possibility of comparing people with disability and those without disability living in the same community though the use of the same indicators. Using people without disability living in the same community as a reference group is necessary in order to disclose inequalities and changes in inequalities when CBR is in place. This is of utmost importance in order to be in line with the United Nations Convention on the Rights of Persons with Disabilities (CRPD), which states that people with disability should have equal rights as everyone else[20]. Finally, since the internationally accepted CBR Guidelines reflect the comprehensive multi-sectoral CBR strategy, indicators based on these guidelines would be the most updated way of monitoring CBR following the five components presented in the CBR Matrix: health, education, livelihood, social and empowerment[15].

**Accounting for this need, WHO initiated a project to develop CBR indicators encompassing health, education, livelihood, social life, and empowerment indicators in accordance with the CBR Matrix.** In the practice of CBR, input and output indicators do not provide an indication of the extent to which a program is achieving its goals or its effectiveness[21], while impacts are long-term effects that are beyond attribution to a CBR intervention as they may reflect broader societal change. Therefore, the CBR indicators were intended to be outcome indicators, as outcomes are the observable short- and intermediate-term changes in a specific group of persons in the CBR area that have been influenced by the outputs. These outcome indicators will serve to standardize monitoring of CBR across areas and countries. The adopted working definition of monitoring was: a descriptive process that provides information on the state of a programme or project at a given time, relative to its respective targets and outcomes[21]. To ensure that different perspectives in terms of CBR expertise were taken into account, and to thereby increase the uptake of the proposed indicators, the entire development was a consensus process in collaboration with the International Disability and Development Consortium (IDDC), stakeholders in the greater CBR community, as well as a team of external researchers.

The aim of this paper is to describe in detail all phases of the development process of the set of global WHO CBR outcome indicators, which were launched by WHO in December 2015. This WHO document, “Community-based rehabilitation indicators manual” (available at http://www.who.int/disabilities/cbr/cbr_indicators_manual/en/[22]), presents the indicators and the recommended data collection and reporting strategy. While the manual is meant to be a “how-to” guide for using the indicators, this manuscript presents the details of the development process for transparency.

## Methods

CBR Guidelines were used as the underlying framework for developing the WHO CBR Indicators because they are internationally accepted as conceptual framework for CBR, were developed together by WHO, IDDC, the United Nations Educational, Scientific and Cultural Organization (UNESCO) and the International Labour Organization (ILO) and are based on a worldwide participatory approach with the involvement of all relevant stakeholders[15]. The CBR Guidelines synthesize global perspectives on CBR and offer recommendations on strengthening the capacity of mainstream and specific services to include people with disability in accessing the benefits of the health, education, livelihood and social sectors and enhance empowerment. For each of these five components of the CBR Matrix (Fig 1), there are five corresponding elements for which the CBR Guidelines present a set of “desirable outcomes” outlining the goals of CBR. Additionally, general overarching desirable outcomes are presented for each component. These desirable outcomes were used as a starting framework for the WHO CBR Indicators. The development process comprised four phases with specific objectives as follows.

### Phase I: Preparatory work

Indicators following the CBR Guidelines’ Matrix have been proposed for monitoring CBR in the past[16]. In addition, different closed and ongoing projects have proposed indicators for disability and health that might match the desirable outcomes proposed in the CBR Guidelines and are in line with the CRPD. To make sure these indicators were taken into account in the present work, previous efforts were systematically scrutinized in Phase I. The specific **objectives of Phase I** were to obtain an overview of the work previously done with the CBR Guidelines as starting point; to obtain an overview of available indicators for disability from other projects; and to study the extent to which these indicators are in line with the CRPD.

**To achieve these goals** an extensive internet search was used to identify disability and population health indicators from initiatives around the world. Indicators from the following projects were included: Human Development Index (http://hdr.undp.org/en/humandev); Millennium Development Goals (http://www.unmillenniumproject.org/goals/); WHO Model Disability Survey (MDS—these indicators were derived directly from the questions, specifically for this study) (http://www.who.int/disabilities/data/mds/en/); UNICEF Multiple Indicator Cluster Survey 4 (UNICEF MICS4) (http://www.unicef.org/statistics/index_24302.html); WHO Global Disability Action Plan; (http://www.who.int/disabilities/actionplan/en/); WHO Core Health Indicators; (http://www.who.int/healthinfo/indicators/2015/en/); and the Zero Project (http://zeroproject.org/indicators-2/). All identified disability and health indicators, as well as the desirable outcomes of the CBR Guidelines were mapped to the CRPD (articles 5 to 30) by two researchers (SA, CM) in order to facilitate comparison.

### Phase II: Framework development

**The objective of Phase II was to use the desirable outcomes published in the CBR Guidelines as a starting point for developing CBR indicators.** Although labelled as “desirable outcomes”, several are formulated rather as output or even impact indicators. In addition, several are dependent on specific CBR objectives, not sensitive to changes at the person level, or not suitable for comparisons across regions. **To achieve this objective** the following three steps were taken:

1. Revising the desirable outcomes to provide a consistent underlying framework for formulating CBR indicators. The revision was a consensus process. Five researchers (hereafter CBR Group) independently categorized each desirable outcome as an input, output, outcome, or impact in accordance with OECD definitions[21]. Modal frequency response analysis was conducted, and where the modal response was not “outcome”, the desirable outcomes were re-formed. This reformation involved a content analysis of the original desirable outcome to formulate it as a true outcome result, expressed at the person level (i.e. “People with disability and their families in the CBR area….”) using an active voice. The individually re-formed desirable outcomes were compiled and the most adequate was selected through an anonymous majority-rule vote. For example the desirable outcome for Health-Assistive Devices originally states: “*Environmental factors are addressed to enable individuals to use their assistive devices in all locations where they are needed*”. The CBR Group unanimously categorized this as an output and voted to reformulate it as “*People with disability use their assistive devices in all areas of the community they need to*”
2. Excluding desirable outcomes that could not be revised to be suitable for cross-sectional and international comparisons using the criteria above. For example, the Empowerment-Political Participation desirable outcome which states “*CBR personnel have increased awareness of the political system*”
3. Selecting the most adequate remaining desirable outcome in terms of feasibility and reliance of information delivered, per component and element of the CBR Matrix. In a two-day workshop the original and re-formed desirable outcomes were presented to IDDC members with CBR expertise. Participants were randomly assigned into two working groups of six persons each. The task was to select or develop one desirable outcome per general component level and one per element of the Matrix, by analysing the content of the desirable outcomes, drawing on field experiences and finally coming to a consensus in the working group. After completing the working groups’ tasks, plenary sessions with all participants took place presenting the original desirable outcomes, CBR Group suggestions, and working groups’ suggestions. The consensus process involved collaborative decision-making with super majority threshold of 75% agreement. This led to the selection of the most adequate desirable outcomes, in terms of coverage of the concepts presented per component and element. For example for Social-Component Level, five desirable outcomes are presented. Two were excluded. Of the remaining three—“*People with disability are valued as members of their families and have a variety of social roles and responsibilities*”, “*People with disability are encouraged and supported to contribute their skills and resources to the development of their communities*”, “*Communities recognize that people with disability are valued members*, *and can make positive contributions to the community*”—the most adequate single formulation was voted to be: “*People with disability feel valued as community members and have a variety of social identities*, *roles and responsibilities*”. When the majority threshold was not reached in the face-to-face meeting, the CBR Group created suggestions which were circulated and edited via email until the majority threshold was met.

### Phase III: Alpha-version of CBR indicators

**Phase III had the objective of developing an alpha-version of CBR indicators and corresponding questions,** along with a sound and simple method for data collection in low resource settings. **To achieve this**, the selected desirable outcomes of Phase II were formulated as proportion indicators at the person level, comparing people with disability to other community members of the same age and gender. To collect data from the indicators, the next step involved developing a survey question for each indicator. Indicators were operationalized into a question or a response option of an overarching question. The use of standardized questions from validated questionnaires or surveys was preferred. When no such standardized question was available, new questions were developed. Questions were proposed by the CBR Group and reviewed by IDDC members in consensus until the majority threshold was reached. Question validation was conducted through pilot implementations. These questions are, however, a suggestion and independent of the indicators: users of the CBR indicators are free to use their own questions to operationalize the indicators. A mobile phone application (app) for android phones was developed to provide an easy-to-use method for data collection (see [22], and https://www.youtube.com/watch?v=NEfJYoGX3uU&t=3s). An interviewer’s manual was prepared for Phase IV (available in [22]).

### Phase IV: Feasibility and validity testing

**Phase IV involved the final selection of the set of WHO CBR Indicators and testing the feasibility and acceptability of using a mobile phone app for data collection.** In order to make data collection as brief as possible, the set of indicators was broken down into two subsets: base indicators which are broad and should be used in all data collection activities to ensure comparability, and supplementary indicators which can provide more specific coverage of the CBR elements and can be selected depending on the specific CBR goals of a program. **This was achieved** through data collection in pilot implementations and an online expert survey in order to determine the relevance of indicators and face validity of questions.

The pilot implementations were carried out in three countries representing three world regions: Guatemala, Egypt and China. Pilots included both persons participating in CBR selected by CBR project managers, and a comparable number of community members without disability matched for age, gender and area of residence for comparison. Interviewers were local CBR staff members, trained by members of the CBR Group in a two day workshop. Since a comparison between boys, girls, men and women was targeted, a gender-balanced convenience sample was recruited and no age restriction was applied. To obtain an overview of unsuitable questions, the distributions of questions’ response options were examined: high proportions of “don’t know” responses were indicative of an underlying problem, and these questions were highlighted as candidates for elimination. To further examine the feasibility and acceptability of the questions, interviewers reported questions they found problematic, for reasons such as the question was confusing, complicated, embarrassing or required follow-up. Questions being marked as problematic in more than 10% of interviews were examined for problems and revised accordingly, while questions with more than 20% were considered for elimination. Additionally, interviewers in Guatemala, the first country running the pilot implementations, participated in focus groups targeting problems regarding conducting interviews and using the app.

The expert survey was internet-based and aimed to gather information on relevance of indicators and validity of questions. Experts working in the field or in CBR research from all six WHO world regions and from varying occupational backgrounds were invited to participate. These experts were all recommended by IDDC. The survey consisted of two parts. After being presented the background of this project, experts were first requested to rank the given list of developed indicators per element of the CBR Matrix in terms of relevance to that element. Second, experts were requested to rate on a scale from 1 (completely adequate) to 5 (not at all adequate), the adequacy of each question as to whether it would retrieve the required information for the indicator. If a question was rated as inadequate, experts were requested to provide feedback and an alternative question.

Results and feedback of the pilot implementations and the expert survey were reviewed by the CBR Group and IDDC, allowing for the selection of a final set of CBR indicators and questions.

## Results

### Phase I: Preparatory work

Of the seven initiatives examined, the WHO Model Disability Survey, WHO Disability Action Plan, and Zero Project present disability-specific indicators. The other projects present general indicators. The most comprehensive coverage of the CRPD and the wide scope of CBR was provided by the MDS (n = 19), UNICEF MICS4 (n = 13), HDI (n = 12), Zero Project (n = 9), MDGs (n = 7), WHO Disability Action Plan (n = 6), and WHO Core Health Indicators (n = 5) (Table 1). The desirable outcomes of the CBR Guidelines covered 23 out of 26 selected CRPD articles.

**Table 1:** Number of indicators from each project which were linked to the CRPD articles.

CRDP Article		CBR desirable outcomes	HDI	MDGs	MDS	UNICEF MICS4	WHO Core Health	WHO Disability Action Plan	Zero Project
**5**	Equality and non-discrimination	1			2				
**6**	Women and disabilities	3	5	3	1	4			
**7**	Children with disabilities		1	4	2	1			
**8**	Awareness-raising	11			25				
**9**	Accessibility	10			22	3		1	12
**10**	Right to life	1				6	5		
**11**	Situations of risk and humanitarian emergencies	1							1
**12**	Equal recognition before the law	6			3				1
**13**	Access to justice	4	1						1
**14**	Liberty and security of the person	1	3						
**15**	Freedom from torture or cruel, inhuman or degrading treatment or punishment	1				3			
**16**	Freedom from exploitation, violence and abuse	1	2			6			
**17**	Protecting the integrity of the person	3				1			
**18**	Liberty of movement and nationality		2		6		1		
**19**	Living independently and being included in the community	29		5	16			2	4
**20**	Personal mobility	8			9	3		1	
**21**	Freedom of expression and opinion, and access to information	8			4	3			6
**22**	Respect for privacy				2				
**23**	Respect for home and the family	4	2	6	20	22	6		1
**24**	Education	33	14	5	20	19			3
**25**	Health	16	5		66	45	38	9	
**26**	Habilitation and rehabilitation	15			43			8	
**27**	Work and employment	18	6	2	28				9
**28**	Adequate standard of living and social protection	7	6	6	18	13	10	1	
**29**	Participation in political and public life	7	1		3				
**30**	Participation in cultural life, recreating, leisure and sport	12			6				
**NUMBER OF ARITCLES COVERED**		**23**	**12**	**7**	**19**	**13**	**5**	**6**	**9**

### Phase II: Revision of CBR desirable outcomes

**Fig 2:**
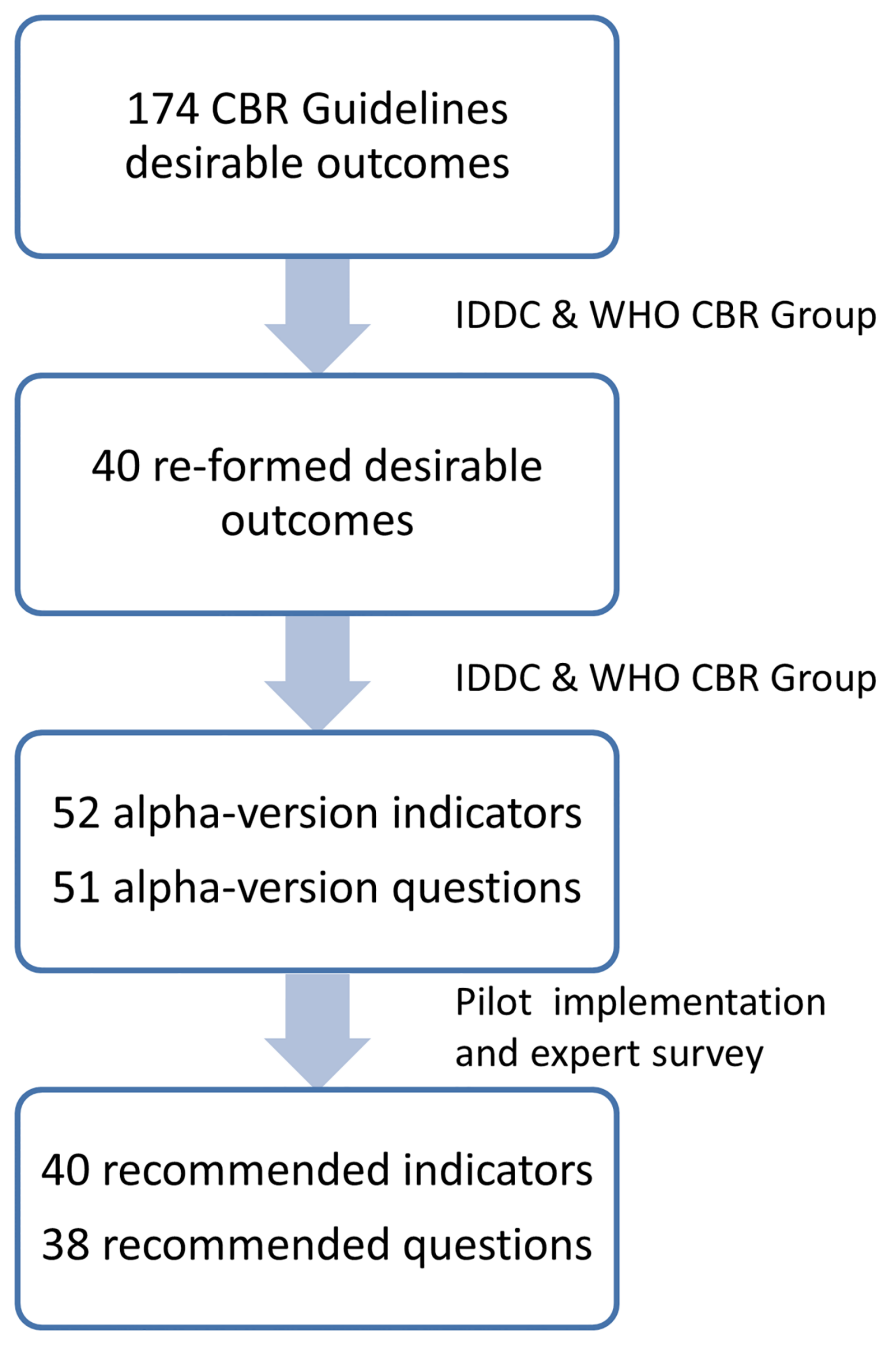
The process beginning with the CBR Guidelines desirable outcomes and leading to the WHO recommended CBR indicators.

Forty-eight of the 174 original desirable outcomes were eliminated for being dependent on specific objectives and activities of CBR, or for not being sensitive to changes at the person level in settings where CBR is carried out. In the components of education and livelihood it was found that some concepts reoccurred throughout the elements. In these cases, the cross-cutting concepts were formulated into single desirable outcomes which were moved to the general component level. For example, in the education component the concept of “*Children*, *youth and adults with disability experiencing equal opportunities to participate in learning opportunities that meet their needs*” reoccurs in all the elements of education, namely early childhood education, primary, secondary, non-formal education and lifelong learning. For this reason this concept was moved to the general component level. As a consequence, primary, secondary and non-formal education no longer had individual desirable outcomes. In livelihood, the concept of “*People with disability earning income through their own chosen economic activities*” reoccurs in the elements of self-employment and wage employment. These were moved to the general component level so that these elements no longer had individual desirable outcomes. Also within livelihood, the element of skills development had overlap with the lifelong learning component of education. For this reason it was decided to incorporate it into lifelong learning. The set agreed on at the end of the consensus process with IDDC consisted of 41 re-formed desirable outcomes (Fig 2).

### Phase III: Alpha set of CBR indicators

Most of the 41 re-formed desirable outcomes (n = 23) were formulated as single indicators; for example “*Men*, *women*, *boys and girls with disability feel they are respected and treated with dignity when receiving health services*” into *“% of people with disability who rate their experience of being treated with respect and dignity by health service providers as good or very good*”. Nine desirable outcomes contained information that was formulated into two indicators; for example “*Men and women with disability access formal and informal social protection measures they need*” into “*% of people with disability who know how to access social protection measures*” and “*% of people with disability who are covered by social protection programs*“. Similarly, two desirable outcomes were formulated into three indicators. Ten desirable outcomes were combined pairwise into single indicators; for example “*Men*, *women*, *boys and girls with disability make use of youth or adult centered learning opportunities to improve their life skills and living conditions*” and “*Men*, *women*, *boys and girls with disability experience equal opportunities to participate in learning opportunities that meet their needs and respect their rights*” were formulated into “*% of people with disability who use life-long learning opportunities to improve their life skills*”. Full formulations are presented in S1 Appendix. It was agreed that differences experienced by men, women, boys and girls would be examined through stratification in the data analysis, and not directly addressed in the formulation of each indicator. A set of 52 alpha-version indicators were operationalized from the 41 re-formed desirable outcomes.

In total, 40 indicators were operationalized into single and four into multiple questions. Eight indicators were operationalized as response option for two overarching questions. At this stage, 51 alpha-version questions were proposed: six originally from and eight adapted from the MDS, three adapted from the UNICEF MICS3, one adapted from the GALLUP Annual Consumption Habits Poll[23], and one from the WHO Quality of Life-BREF[24]. The remaining 32 questions were developed by the CBR Group and reviewed by IDDC members in a consensus process (see S1 Appendix). This alpha-version contained 52 indicators and 51 corresponding questions that were then implemented in the app.

### Phase IV: Feasibility and validity testing

The total sample of the pilot implementations consisted of 801 participants, 53.4% female, with a mean age of 29.6 (SD 21.3). Further characteristics of participants are reported in Table 2.

**Table 2:** Descriptive statistics of the 801 pilot implementation participants.

	China N (%)		Egypt N (%)		Guatemala N (%)		Total N (%)	
	Control (n = 132)	People with disability (n = 128)	Control (n = 118)	People with disability (n = 119)	Control (n = 160)	People with disability (n = 143)	Control (n = 406)	People with disability (n = 395)
**Gender (female)**	74 (57.8)	70 (53.0)	65 (55.1)	55 (46.2)	102 (63.8)	62 (43.4)	241 (59.4)	187 (47.5)
**Proxys used**	0	5 (3.8)	38 (32.2)	59 (49.6)	77 (49.0)	109 (76.8)	115 (28.6)	173 (44.1)
**Children**	5 (3.9)	13 (9.9)	36 (30.5)	43 (36.4)	76 (48.4)	93 (66.0)	143 (35.2)	163 (41.4)
**Mean age (SD)**	42.1 (±19.0)	46.0 (±20.4)	31.1 (±20.4)	31.2 (±21.4)	17.4 (±11.9)	13.6 (±10.9)	29.5 (±20.2)	29.8 (±22.4)

Nineteen questions (37.3%) were reported as problematic in more than 10% of interviews, with five questions (9.8%) reported as problematic in more than 20% of interviews. Main problems reported by interviewers were that the question was confusing or difficult to understand, or that the question needed follow-up or clarification. Most problems were reported in Egypt (70.3%), followed by Guatemala (27.5%), and then China (2.2%). Focus groups in Guatemala, the first country carrying out the implementation, revealed problems that were then addressed before the pilots in Egypt and China. An overarching problem was the high complexity of some questions, and difficulties with the response options, which ranged from 5(completely) to 1(not at all). For this reason, response options were re-ordered from 1(not at all) to 5(completely), and show cards were used for visual representations in Egypt and China.

The expert survey invited 72 experts to participate, with 31 completing the survey. The majority of participants were male (54.8%) and worked in NGOs or INGOs (61.3%), while those who had worked in the field of CBR for 20 years or more (35.5%) and those working in the South-East Asia Region (38.7%) represented the largest proportions of respondents (Table 3). Per component and element, the indicator ranked as the most relevant by the majority of experts was selected. In cases where no indicator reached a majority vote, the top indicators were examined and selected by the CBR Group.

**Table 3:** Demographic characteristics of the 31 expert survey participants.

		N (%)
**Gender**	Female	14 (45.2%)
**Age**	under 40	8 (25.8%)
	40–59	15 (48.4%)
	60+	8 (25.8%)
**Time period spent working in the field of CBR**	Under 10 years	13 (41.9%)
	10–19 years	7 (22.6%)
	20+ years	11 (35.5%)
**Primary world region of work**	African Region	5 (16.1%)
	Region of the Americas	2 (6.5%)
	South-East Asia Region	12 (38.7%)
	European Region	1 (3.2%)
	Eastern Mediterranean Region	3 (9.7%)
	Western Pacific Region	5 (16.1%)
	Global	3 (9.7%)
**Primary working position**	Academia	11 (35.5%)
	DPO	1 (3.2%)
	NGO/INGO	19 (61.3%)
	Government	2 (6.5%)
	Other	6 (19.4%)

The analysis of the results of the survey and the pilot implementations led to the selection 40 CBR indicators[22]. The indicators are broken down into two sets: 13 base and 27 supplementary indicators. Base indicators are broad enough to capture the situation of people with disability, independent of specific CBR activities carried out and are derived from the general component level desirable outcomes. These indicators are recommended to be included in any data collection. All but one of the component level desirable outcomes was selected as a base indicator. The exception was the general livelihood indicator of “*People with disability get to make their own decisions about how to use their money*”. The CBR Group saw that the concept of “*People with disability having enough money to meet their needs*” was not covered by any indicator, and therefore created this as a base indicator, with the initial indicator remaining as a supplementary indicator. Supplementary indicators provide more specific coverage of the CBR elements and can be selected depending on the specific goals and strategies of a program. Base CBR indicators have eight corresponding questions and supplementary have 30 corresponding questions. The WHO manual presents the full set of indicators and the data collection procedures[22].

## Discussion

**The aim of this paper was to describe in detail all phases of the development process of a recently launched set of global CBR outcome indicators, based on the CBR Guidelines, which are suitable to monitor CBR.** The proposed set of indicators includes 13 base and 27 supplementary CBR indicators, is grounded on the internationally acknowledged CBR Guidelines, and is the result of a collaborative, consensus-orientated and evidence-based effort between WHO, IDDC and the broader CBR community. These indicators will serve to capture the situation of people with disability in settings where CBR is carried out, independent of the specific objectives and implemented activities of a program. These indicators will support WHO and member states in their efforts towards strengthening CBR, as requested in the *Global Disability Action Plan*, through generating evidence on the effectiveness of CBR [6]. The use of the proposed CBR indicators will generate the evidence needed by NGO’s, DPO’s, and the broad community involved in CBR to advocate for broader and integrated CBR implementation in different settings, including at the national level.

**The use of the CBR Guidelines as a multi-sectorial reference framework for the CBR indicators is essential. Due to the heterogeneity and varying contexts in which CBR is implemented, an appropriate framework is needed as a basis for the monitoring process**[4,7]. The CBR Guidelines and the corresponding desirable outcomes were selected as a framework for the proposed CBR indicators as they encompass a unified understanding of CBR concepts in line with the CRPD[9,25]. Though a global set was previously suggested[13,26], there is some disagreement as to whether a global set of indicators, even when based on the CBR Guidelines and the corresponding matrix, can cover the cultural and methodological diversity of CBR[18,19]. To account for this, the CBR indicators proposed here take advantage of the several elements of each CBR Matrix component and use them to offer a possibility of customizing data collection. Stakeholders responsible for data collection are requested to use the 13 base CBR indicators in all data collection to guarantee standardisation and comparability. However, additional indicators can be selected out of the 27 supplementary CBR indicators so that the data collection can be shaped to monitor more specific programs’ goals, cultural settings, or requirements of funding bodies. In summary, the indicators presented in this project combine the advantage of providing a means of collecting global data for cross-program comparisons, while also addressing the diversity of CBR by allowing the flexibility to customize data collection.

**The flexibility presented in indicator selection and the corresponding mobile phone app help to encourage the uptake of the CBR indicators by making data collection as quick and simple as possible.** Providing intuitive procedures to customize and carry out data collection allows data collection to be carried out by any community member, which is in line with suggestions that the monitoring process should involve community members and people with disability to allow for engagement of the local community, thereby fostering greater community ownership and sustainability[9]. The app is free to download on Google Play and works offline. Interviews using base indicators can be completed within five minutes. Interviews are either submitted to a selected e-mail address or anonymously to a central and secure server located at WHO upon acceptance of the data protection agreement on the phone. Furthermore, in order to increase the motivation for data collection, if completed interviews are submitted to the central server, the data will be organized so that the indicator results can be presented as diagrams. These diagrams will be able to show the differences between people with disability and those without disability in the community surveyed, and within those groups, the differences between boys, girls, men and women. In case stakeholders are willing to share the data with WHO and the CBR community, anonymous comparisons of different programs and regions will be implemented in the CBR page of WHO’s website.

**The CBR indicators proposed at present are the first necessary step towards the global monitoring and evaluation of CBR.** They focus on monitoring and on outcomes at the individual level with the results from each indicator allowing for the identification of discrepancies experienced by people with disability. For example, when the indicator *“% of people with disability who acquire education in mainstream education facilities*” presents low percentage it can indicate exclusion of people with disability from their peers. These results can be further interpreted to see the effects on the community members as a whole. The next step in the monitoring process of CBR is to broaden the perspective by developing system indicators suitable to capture societal, administrative, attitudinal, and environment changes. Further work is also needed to develop sound and reliable indicators for the evaluation of CBR, in terms of creating making systematic judgements regarding the relevance, fulfilment of objectives, efficiency, effectiveness, impact, and sustainability of CBR[21]. As CBR is a continuous process there is an urgent need for longitudinal data to capture change over time, both for monitoring and evaluation, which will come through follow-up data collection.

**Some limitations that come as an inherent result of using indicators, as is the case for the CBR indicators, should be mentioned here**. People may argue that indicators have been shown to lead to over-aggregation and over simplification of data while only measuring what is quantifiable, and not always match to what is important to people[27]. Being unaware of this can lead to overconfidence in the relevance of the data collected, and thereby lead to incompleteness in the overview the indicators should generate. Furthermore, data gathered with indicators should be complemented with data from direct experience if an in-depth understanding is targeted, which can only be collected through qualitative approaches[27]. These facts might result in reluctance to use the CBR indicators. It is important to stress, however, that until now qualitative studies have dominated the field of CBR, and that despite all research carried out, recent reviews continue to stress the lack of evidence on the effectiveness of CBR[4]. In this sense, the proposed indicators may suffer from the inherent shortcomings of indicators, but they provide a unique opportunity to collect standardized global data on CBR after more than 30 years of attempts to do so. Data collected with the indicators, combined with results from available qualitative work, could finally prove what is strongly assumed, namely that CBR is effective and worth the effort required for implementation.

**Finally, it is important to stress that the *Global Disability Action Plan* explicitly calls for the strengthening of CBR through monitoring and evaluation**[6]. It is strongly recommend that qualitative work on disclosing potential sector, country, regional or cultural barriers, as usually done in implementation research, be carried out alongside the first implementations of the CBR indicators. Researchers and stakeholders are encouraged learn from data collection efforts and to contribute to the further development of strategies that can guarantee uptake of the CBR indicators. Users of the CBR indicators proposed here are therefore called to be active participants in achieving this goal by periodically collecting data, by reporting their experiences during data collection and by sharing data with WHO and the CBR community. This will contribute to the creation of a strong evidence base that can ultimately deliver arguments to improve CBR and potentially advocate for broader and more sustainable implementation.

## Conclusion

The use of the CBR indicators proposed in this work and corresponding questions allow for reliable, easy and comparable data collection to demonstrate the effect of CBR, and thereby potentially broaden the appeal for its implementation. These indicators capture the situation of people with disability in comparison to other community members in the aspects of health, education, social life, livelihood and empowerment, as outlined in the CBR Guidelines[15]. When data is collected over time in a community it will capture changes in the lives of people with disability, as well as support monitoring of the implementation of the CRPD at the community level in an easy and efficient way. These indicators allow for further comparability across settings and countries. The CBR indicators are understood as a starting point towards generating sound and standardised evidence for CBR. Further work is needed to complement these indicators with system level indicators tackling factors in the environment, to identify barriers that might prevent their uptake, and to develop methods of using the generated information in economic evaluations of CBR.

## Supporting information

S1 Appendix Revised desirable outcomes and the corresponding alpha-version of indicators and questions resulting from the IDDC consultation. (DOCX)

## References

[1] International Labour Organization, United Nations Educational, Scientific and Cultural Organization and the World Health Organization. CBR: A strategy for rehabilitation, equalization of opportunities, poverty reduction and social inclusion of people with disabilities: joint position paper. Geneva: World Health Organization; 2004.

[2] Kuipers P, Allen O. Preliminary guidelines for the implementation of Community Based Rehabilitation (CBR) approaches in rural, remote and Indigenous communities in Australia. Rural and Remote Health. 2004;4(2004):291. 15885015

[3] Kendall Elizabeth B N, Larner Joanne. Community-based service delivery in rehabilitation: the promise and the paradox. Disability & Rehabilitation. 2000;22(10):435–45. 10950496 10.1080/09638280050045901

[4] Iemmi V, Gibson L, Blanchet K, Suresh Kumar K, Rath S, Hartley S, et al Community-based rehabilitation for people with disabilities in low-and middle-income countries: a systematic review. Campbell Systematic Reviews. 2015;15.

[5] World Health Organization & World Bank. World report on disability. Geneva: World Health Organization; 2011.

[6] World Health Organization. WHO global disability action plan 2014–2021: better health for all people with disability. Geneva: World Health Organization; 2014.

[7] Lukersmith S, Hartley S, Kuipers P, Madden R, Llewellyn G, Dune T. Community-based rehabilitation (CBR) monitoring and evaluation methods and tools: a literature review. Disability and rehabilitation. 2013;35(23):1941–53. 10.3109/09638288.2013.770078 23574396

[8] Cornielje H, Velema JP, Finkenflugel H. Community based rehabilitation programmes: Monitoring and evaluation in order to measure results. Leprosy Review. 2008;79(1):36–49. 18540236

[9] Grandisson M, Hébert M, Thibeault R. A systematic review on how to conduct evaluations in community-based rehabilitation. Disability and rehabilitation. 2014;36(4):265–75. 10.3109/09638288.2013.785602 23614357 PMC3913006

[10] Sharma M. Evaluation in community based rehabilitation programmes: a strengths, weaknesses, opportunities and threats analysis. Asia Pacific Disability Rehabilitation Journal. 2007;18(1):46–62.

[11] Workshop on Community-Based Rehabilitation and Country Experiences of CBR; 1996 Jan; Bologna, Italy. Cornell University IRL School: 1996 [cited 2016 Jan 20]. http://digitalcommons.ilr.cornell.edu/cgi/viewcontent.cgi?article=1155&context=gladnetcollect.

[12] Velema J. Making sense of rehabilitation projects: classification by objectives. Leprosy Review. 2000;71:472–85. 11201902 10.5935/0305-7518.20000049

[13] Wirz S, Thomas M. Evaluation of community-based rehabilitation programmes: a search for appropriate indicators. International journal of rehabilitation research. 2002;25(3):163–71. 12352169 10.1097/00004356-200209000-00001

[14] Kuipers P, Quinn R. The template: A cooperative approach to evaluating community rehabilitation services. Journal of Rehabilitation. 2003;69(1):4.

[15] World Health Organization, United Nations Educational, Scientific and Cultural Organization, & International Labour Organization. Community-based rehabilitation: CBR Guidelines. Geneva: World Health Organization; 2010.

[16] Robb A, editor. Report on “Technical Meeting on Development of CBR M&E” and the 1st CBR World Congress; 2012 Nov 26–28; Agra, India. 2012.

[17] Madden RH, Lukersmith S, Millington M J, Scarf C, Fortune N, Hartley S, Llewellyn G. Participatory Monitoring of Community-Based Rehabilitation and other Disability-Inclusive Development Programmes: the Development of a Manual and Menu. Disability, CBR & Inclusive Development. 2016; 26(4):26–52.

[18] Adeoye A, Seeley J, Hartley S. Developing a tool for evaluating community-based rehabilitation in Uganda. Disability and rehabilitation. 2011;33(13–14):1110–24. 10.3109/09638288.2010.521613 20929422

[19] chung EYin-han, Packer TL, Yau M. A framework for evaluating community-based rehabilitation programmes in Chinese communities. Disability and rehabilitation. 2011;33(17–18):1668–82. 10.3109/09638288.2010.541545 21171842

[20] The United Nations (2006). Convention on the Rights of Persons with Disabilities Treaty Series, 2515, 3. 10.1515/9783110208856.203 18348362

[21] Organisation for Economic Co-operation and Development (2004). Glossary of Key Terms in Evaluation and Results Based Management. Paris: Organisation for Economic Co-operation and Development.

[22] World Health Organisation, International Disability and Development Consortium (2015). Capturing the difference we make: Community-based rehabilitation indicators manual. Geneva: WHO.

[23] GALLUP (2013). Poll Social Series: Consumption Habits. Washington, D.C.: GALLUP.

[24] World Health Organization (2012). WHO quality of life-bref (WHOQOL-BREF). Geneva: World Health Organization.

[25] Thomas M. Reflections on community-based rehabilitation. Psychology & Developing Societies. 2011;23(2):277–91.

[26] Finkenflügel H, Cornielje H, Velema J. The use of classification models in the evaluation of CBR programmes. Disability and rehabilitation. 2008;30(5):348–54. 10.1080/09638280701257288 17852305

[27] Meadows D (1998). Indicators and Information Systems for Sustainable Development. Hartland, VT: Sustainability Institute.

