# Supplementary material for: Development of indicators for monitoring Community-Based Rehabilitation
Source: PLoS One. 2017 Jun 2;12(6):e0178418. doi: 10.1371/journal.pone.0178418 (PMC5456090; doi:10.1371/journal.pone.0178418)
Supplement: S1 Appendix — (DOCX) [file pone.0178418.s001.docx]

Appendix 1: Revised desirable outcomes and the corresponding alpha-version of indicators and questions resulting from the IDDC consultation

| **HEALTH COMPONENT** | | | |
| --- | --- | --- | --- |
| **CBR Guidelines Element** | **Revised Desirable Outcome** | **Indicator** | **Question** |
| General | Men, women, boys and girls with disability equally access health services and engage in activities needed to achieve the highest attainable standard of health | % of people with disability who rate their health as good or very good | I will start with a question about your overall health, including your physical and your mental health: In general, how would you rate your health today? 1=Very good; 2=Good; 3=Neither poor nor good; 4=Poor; 5=Very poor |
| General | Men, women, boys and girls with disability feel they are respected and treated with dignity when receiving health services | % of people with disability who rate their experience of being treated with respect and dignity by health service providers as good or very good | On your last visit to a health care provider, to what extent are you satisfied with the level of respect you were treated with?  1=Not at all; 2=A little; 3=Moderately; 4=Mostly; 5=Completely |
| Promotion | Men, women, boys and girls with disability know how to achieve good levels of health and participate in activities contributing to their health | % of people with disability and their families that know (aware of) that physical activity and eating habits influence their health | Has your (doctor, CBR worker, or any other health professional) ever discussed with you the benefits of eating a healthy diet, engaging in regular physical exercise, or not smoking? 1=Yes; 2=No |
| Prevention | Men, women, boys and girls with disability participate in activities that prevent them and future generations from getting ill | % of people with disability who receive full immunization as recommended for their country by WHO | When was the last time you have been vaccinated? 1 = In the last 5 years; 2 = In the last 5-10 years; 3 = Longer than 10 years; 4 = Never |
| Prevention |  | % of children with disability who receive full immunization as recommended for their country by WHO | When was the last time [NAME] was vaccinated? 1 = In the last 2 years; 2 = More than 2 years; 3 = Never |
| Prevention |  | % of children with disability who receive the recommended health check-ups | When was the last time [NAME] had a health check-up? 1 = In the last year; 2 = Between 1-2 years ago; 3 = Between 3-5 years ago; 4 = Longer than 5 years ago; 5 = Never |
| Prevention | Decision makers and community actively engage in reducing health conditions | % of people with disability who live in communities where decision makers actively engage in reducing health conditions | To what extent have people in your community done anything to make your neighbourhood a cleaner, healthier, and safer place to live? 1=Not at all; 2=A little; 3=Moderately; 4=Mostly; 5=Completely |
| Medical Care | Men, women, boys and girls with disability access and benefit from quality medical services appropriate to their life stage needs and priorities | % of people with disability that needed medical care in the last 12 months and did not get the care they need | In the last 12 months, has there been a time when you needed health care but did not get that care? 1=Yes; 2=No |
| Medical Care |  | % of people with disability and their families that have access to medical care | Which reason(s) best explain why you did not get health care? 1=Health care facility too far away; 2=Could not afford the cost of the visit; 3=No transport available; 4=Transport not accessible; 5=Could not afford the cost of transport; 6=Were previously badly treated; 7=Could not take time off work or had other commitments; 8=Health care provider's drugs or equipment were inadequate; 9=Health care provider's skills were inadequate; 10=Did not know where to go; 11=Tried but were denied health care; 12=Thought you were not sick enough; 13=Other |
| Medical Care |  | % of people with disability that have the experience of being involved in making decisions for their treatment | On your last visit to a health care provider, to what extent were you involved in making decisions for your treatment? 1=Not at all; 2=A little; 3=Moderately; 4=Mostly; 5=Completely |
| Rehabilitation | Men, women, boys and girls with disability engage in planning and carry out rehabilitation activities with the required services | % of people with disability that needed rehabilitation services in the last 12 months and did not get the services they need | In the last 12 months, has there been a time when you needed rehabilitation services, such as physical, occupational, or speech therapy, but did not get those services? 1=Yes; 2=No |
| Rehabilitation |  | % of people with disability that have access to rehabilitation services | Which reason(s) best explains why you did not get that rehabilitation service? 1=Rehabilitation facility too far away; 2=Could not afford the cost of the visit; 3=No transport available; 4=Transport not accessible; 5=Could not afford the cost of transport; 6=Were previously badly treated; 7=Could not take time off work or had other commitments; 8=The rehabilitation service provider's drugs or equipment were inadequate; 9=The rehabilitation service provider's skills were inadequate; 10=Did not know where to go; 11=Tried but were denied health care; 12=Thought you were not sick enough; 13=Other |
| Assistive Devices | Men, women, boys and girls with disability have access to, use, and know how to maintain appropriate assistive products in their daily life | % of people with disability that have access to assistive products appropriate to their needs | Do you use any aids to help you get around such as cane, crutch, or wheelchair; or to help you with self-care such as grasping bars, hand, or arm brace?  1=Yes, and it works well; 2=Yes, but it doesn't work or isn't appropriate; 3=No, but I need it; 4=No, because it's broken or not appropriate; 5=No, I don't need it |
| Assistive Devices |  |  | Do you use anything to help you to see better, such as glasses? 1=Yes, and it works well; 2=Yes, but it doesn't work or isn't appropriate; 3=No, but I need it; 4=No, because it's broken or not appropriate; 5=No, I don't need it |
| Assistive Devices |  |  | Do you use anything to help you hear or communicate better? 1=Yes, and it works well; 2=Yes, but it doesn't work or isn't appropriate; 3=No, but I need it; 4=No, because it's broken or not appropriate; 5=No, I don't need it |
| Assistive Devices |  | % of people with disability using assistive devices that know how to maintain them | Do you know how to keep your assistive device in good working condition? 1=Yes; 2=No |

| **EDUCATION COMPONENT** | | | |
| --- | --- | --- | --- |
| **CBR Guidelines Element** | **Revised Desirable Outcome** | **Indicator** | **Question** |
| General | Policies and resources are conducive to education for people with disability and ensure smooth transitions through different stages of learning   Children with disability participate in and complete quality primary education in an enabling and supportive environment  Men, women, boys and girls with disability have resources and support to enroll and complete quality secondary and higher education in an enabling and supportive environment  Youth with disability experience post school options on an equal basis with their peers | % of youth with disability that have completed secondary education before age 20  % of youth with disability who are attending secondary education  % of people with disability who are attending or have completed higher education  % of people with disability who have educational or vocational options after obtaining their educational certificate or degree  % of people with disability who have professional training | What is the highest level of education that you have achieved? If currently receiving education:  What is the grade that you are currently working towards completing?  1=No schooling or never completed any grade; 2=Elementary education; 3=Vocational education; 4=Professional training; 5=Secondary school; 6=College; 7=University; 8=Post-graduate studies; 9=Other |
| General |  | % of people with disability who acquire education in mainstream education facilities | Where did/do you receive your education? 1=Regular institutions; 2=Specialized institutions; 3=Home-schooling; 4=Other forms of education |
| General | Children and youth with disability participate in a variety of non-formal learning opportunities based on their needs and desires | % of people with disability who participate in learning opportunities that meet their needs | To what extent does your education contribute to achieving your goals? 1=Not at all; 2=A little; 3=Moderately; 4=Mostly; 5=Completely |
| General |  | % of people with disability study in enabling and supportive environment | To what extent were/are you included and accepted by your teachers and peers?  1=Not at all; 2=A little; 3=Moderately; 4=Mostly; 5=Completely |
| Early Childhood | Children with disability actively participate in early childhood developmental activities and play, either in a formal or informal environment | % of children with disability age 36-59 months who are participating in early childhood education activities | Does [NAME] attend any organized learning or early childhood education programme, such as a private or government facility, including kindergarten or community child care? 1=Yes; 2=No |
| Lifelong Learning | Men, women, boys and girls with disability make use of youth or adult centered learning opportunities to improve their life skills and living conditions | % of people with disability who use life-long learning opportunities to improve their life skills | Do you participate in learning opportunities to improve your skills for everyday life or work? 1=Yes; 2=No |
| Lifelong Learning | Men, women, boys and girls with disability experience equal opportunities to participate in learning opportunities that meet their needs and respect their rights |  | To what extent does it fit your needs? 1=Not at all; 2=A little; 3=Moderately; 4=Mostly; 5=Completely |

| **LIVELIHOOD COMPONENT** | | | |
| --- | --- | --- | --- |
| **CBR Guidelines Element** | **Revised Desirable Outcome** | **Indicator** | **Question** |
| General | Men and women with disability have paid and decent work in the formal and informal sector on equal bases with others  Women and men with disability earn income through their own chosen economic activities  Youth and adults with disability acquire marketable skills on an equal basis with others through a range of inclusive training opportunities | % of people with disability who are self-employed or own-account workers  % of people with disability who are working for wages or salary with an employer | What is your current working situation? 1=Not working and looking for work; 2=Not working and not looking for work (for example student or housewife.); 3=Working for wages or salary with an employer (full- or part-time); 4=Working for wages, but currently on sick leave for more than three months; 5=Self-employed or own-account worker; 6=Working as unpaid family member (e.g. working in family business); 7=Retired because of the health condition; 8=Retired due to age; 9=Early retirement; 10=Other |
| General | Inclusive policies, practices and appropriate resources, defined with PwD, enable equal participation of women and men with disability in livelihood (training, finance, work opportunities and social protection) | % of people with disability who are involved in developing inclusive policies and practices for equal participation in the labour sector | Do you engage in local or national organizations working towards disability inclusive working conditions? 1=Yes; 2=No |
| General | Women and men have control over the money they earn | % of people with disability who get to make decisions of how to use his/her money | Do you get to decide how to use your money?  1=Not at all; 2=A little; 3=Moderately; 4=Mostly; 5=Completely |
| Financial Services | Men and women with disability have access to grants, loans and other financial services on an equal basis with others | % of people with disability who know how to access financial services | Should you need financial services such as credit, insurance, grants, savings programs, would you know how to get them? 1=Yes; 2=No |
| Financial Services | Men and women with disability participate in local saving and credit schemes | % of people with disability who use financial services such as grants and loans | Do you currently have any credit, insurance, grants, or savings programs related to your work?  1=Yes; 2=No |
| Social Protection | Men and women with disability access formal and informal social protection measures they need | % of people with disability who know how to access social protection measures | Should you need social protection against loss of income through old age, sickness or disability, would you know how to receive it?  1=Yes; 2=No |
| Social Protection |  | % of people with disability who are covered by social protection programs | Do you currently benefit from any social protection program? 1=Yes; 2=No |

| **SOCIAL COMPONENT** | | | |
| --- | --- | --- | --- |
| **CBR Guidelines Element** | **Revised Desirable Outcome** | **Indicator** | **Question** |
| General | Men, women, boys and girls with disability feel valued as community members and have a variety of social identities, roles and responsibilities | % of people with disability that feel valued as individuals by members of their community | Do you feel that other people respect you? For example, do you feel that others value you as a person and listen to what you have to say? 1=Not at all; 2=A little; 3=Moderately; 4=Mostly; 5=Completely |
| General | Men, women, boys and girls with disability feel safe in their family and community | % of people with disability who feel safe in their family and community | Do you feel safe in your everyday life? 1=Not at all; 2=A little; 3=Moderately; 4=Mostly; 5=Completely |
| Personal Assistance | Men, women, boys and girls with disability access and control the way needed personal assistance is provided | % of people with disability who get to make their own decisions about the personal assistance they need | Do you get to make decisions about the personal assistance that you need (who assists you, what type of assistance, when to get assistance)? 1=Not at all; 2=A little; 3=Moderately; 4=Mostly; 5=Completely |
| Relationships, Marriage, and Family | Men, women, boys and girls with disability experience support of the community and their families to socialize and form age-appropriate and respectful relationships | % of people with disability who get to make their own decisions about their personal relationships | Do you get to make your own decisions about your personal relationships, including family, friends and coworkers?  1=Not at all; 2=A little; 3=Moderately; 4=Mostly; 5=Completely |
| Relationships, Marriage, and Family |  | % of people with disability who feel respected in their decisions regarding personal relationships | And to what extent do you feel the people around you respect these decisions? 1=Not at all; 2=A little; 3=Moderately; 4=Mostly; 5=Completely |
| Culture and Arts | Men, women, boys and girls with disability participate in artistic, cultural or religious events in and outside their home as they choose | % of people with disability who get to participate in artistic, cultural or religious activities | Do you get to participate in artistic, cultural or religious activities? 1=Not at all; 2=A little; 3=Moderately; 4=Mostly; 5=Completely |
| Recreation, Leisure, and Sports | Men, women, boys and girls with disability participate in inclusive or specific recreation, leisure and sports activities | % of people with disability who get to participate in mainstream recreational, leisure and sports activities | Do you get to participate in community recreational, leisure and sports activities?  1=Not at all; 2=A little; 3=Moderately; 4=Mostly; 5=Completely |
| Recreation, Leisure, and Sports |  | % of people with disability who get to participate in recreational, leisure and sports activities for people with special needs | To what extent are the recreational, leisure and sports activities adapted to suit your needs? 1=Not at all; 2=A little; 3=Moderately; 4=Mostly; 5=Completely |
| Justice | All PwD are recognized as equal citizens with legal capacity | % of people with disability who know their legal rights | To what extent do you know your legal rights? 1=Not at all; 2=A little; 3=Moderately; 4=Mostly; 5=Completely |
| Justice | PwD access and use formal and informal mechanisms of justice | % of people with disability who know how to access mechanisms of justice | Should you need to access the justice system, would you know how to? 1=Yes; 2=No |
| Justice |  | % of people with disability who use mechanisms of justice | Do you currently make use of formal or informal forms of justice?  1=Yes; 2=No |
| Justice |  | % of people with disability that are satisfied with the level of respect and dignity that they receive from the stakeholders in the justice sector | To what extent are you satisfied with the level of respect you are treated with by people working in the formal and informal justice system, such as police officers, lawyers, judges, or any other justice authority in the community?  1=Not at all; 2=A little; 3=Moderately; 4=Mostly; 5=Completely |

| **EMPOWERMENT COMPONENT** | | | |
| --- | --- | --- | --- |
| **CBR Guidelines Element** | **Revised Desirable Outcome** | **Indicator** | **Question** |
| General | PwD make informed choices and decisions | % of people with disability who get to make informed choices and decisions | Do you get to make the big decisions in your life? For example, deciding who to live with, where to live, or how to spend your money? 1=Not at all; 2=A little; 3=Moderately; 4=Mostly; 5=Completely |
| General | PwD advocate for and or exercise their rights | % of people with disability who know and exercise their rights | Do you think that the laws and policies in your country provide people with disability equal rights as other people? 1=Not at all; 2=A little; 3=Moderately; 4=Mostly; 5=Completely |
| General |  |  | Should your rights (such as accessing education or voting) be denied or violated would you know what to do? 1=Yes; 2=No |
| Advocacy and Communication | Men, women, boys and girls with disability effectively use communication skills and resources (including supportive decision making) to facilitate interactions and influence change | % of people with disability who have the communication skills to express their wishes and objections effectively | Are you satisfied with your ability to communicate with other people? For example, how you say things or get your point across 1=Not at all; 2=A little; 3=Moderately; 4=Mostly; 5=Completely |
| Community Mobilization | Men, women, boys and girls with disability play a catalyzing role in mobilizing key community stakeholders to create an enabling environment | % of people with disability who have a role in shaping their communities to achieve equal opportunities for all | Do you get to influence the way your community responds to the needs and rights of people with disability? 1=Not at all; 2=A little; 3=Moderately; 4=Mostly; 5=Completely |
| Political Participation | Men and women with disability participate in political processes on an equal basis with others | % of people with disability who engage in local or national politics and in civil society organizations | Did you vote in the last election?  1=Yes; 2=No |
| Self-Help Groups | PwD actively engage in and benefit from self-help groups in the local communities, if they choose (inclusive or specific) | % of people with disability who actively engage in and benefit from self-help groups | Are you a member of a self-help group?  1=Yes; 2=No, but I would like to; 3=No, I don't want to |
| Self-Help Groups | Self-help groups come together to form federations to harness collective energy and influence positive change | % of people with disability who are members of self-help groups which are part of a larger federation | Is your self-help group a member of a federation of self-help groups? 1=Yes; 2=No |
| Disabled People’s Organizations | Men and women with different kinds of disability living in different situations (rural or urban areas, poor or rich, refugees) feel they are adequately represented by DPO  DPOs are influential stakeholders in decision-making | % of people with disability who feel that they are adequately represented by DPO’s | To what extent do you feel Disabled Peoples Organizations adequately represent your concerns and priorities? 1=Not at all; 2=A little; 3=Moderately; 4=Mostly; 5=Completely |

*taken or adapted from the MDS

**adapted from the UNICEF MICS3 Questionnaire for Children Under Five

+adapted from the GALLUP annual Consumption Habits poll

++adapted from the WHO Quality of Life-BREF
